# Why does higher education sometimes lead to unhappiness in China? An explanation from housing assets

**DOI:** 10.3389/fpsyg.2022.1002647

**Published:** 2022-10-20

**Authors:** Yidong Wu, Renjie Zhao, Yalin Zhang, Zhuo Chen

**Affiliations:** ^1^School of Business, Anhui University of Technology, Ma'anshan, China; ^2^School of Economics and Management, Northwest University, Xi'an, China; ^3^School of Economics, Nanjing University of Posts and Telecommunications, Nanjing, China; ^4^School of Government Audit, Nanjing Audit University, Nanjing, China

**Keywords:** higher education, happiness, housing asset, housing price, China

## Abstract

This article aims to answer the question that whether higher education would lead to happier life in China and tries to provide some explanations from the perspective of housing asset. Using data from four waves of China Household Finance Survey (CHFS), we find that higher education on average is significantly negatively correlated with people's happiness in urban China. Higher education tends to prevent people from achieving “extremely happy” lives; instead, it is more likely to lead to “acceptable” lives. Based on the realities of housing market in urban China, we find that housing asset plays the mediating role in the relationship between higher education and happiness. Specifically, years of schooling could evidently compress the years of being homeowners; as a result, highly educated people generally have more unpaid housing debts and bear more housing purchase costs due to the soaring housing prices. Meanwhile, higher education has negative effect on people's happiness in cities with relatively high housing prices, while this effect is insignificant in cities with relatively low housing prices. Moreover, the market-oriented housing reform that launched in 1998 has negative impact on highly educated people's happiness, since it has dramatically boosted housing prices and essentially changed housing distribution system for urban employees. Besides, we also find that Ph.D graduates are the relatively unhappiest people compared to bachelors or masters. Obviously, our findings have important policy implications for Chinese government to understand and resolve the “education-happiness paradox.”

## Introduction

Happiness is the only rational goal of life and the only ultimate objective of public policy (Ng and Ho, 2006). There is a large body of literature trying to explain happiness and investigate what factors relate to people's happiness status or subjective wellbeing (Easterlin, 1974, 2003; Diener et al., 1999; Frey and Stutzer, 2002; Diener, 2009; Steiner et al., 2010; Myers and Diener, 2013; Asadullah et al., 2018; Berggren et al., 2018; Clark et al., 2019). Especially, education level is generally confirmed to play a positive role in one's perceived happiness (Smyth et al., 2010; Chen, 2012; Cuñado and Gracia, 2012; Nikolaev and Rusakov, 2016). Besides, education is always regarded as a positive factor in various indexes to measure people's wellbeing. For example, the United Nations Development Programme (UNDP) has developed Human Development Index (HDI) since the 1990s, one of the three key dimensions (i.e., a long and healthy life, access to education, and a decent standard of living) of HDI is access to education, which measured by expected years of schooling of children at school-entry age and mean years of schooling of the adult population^1^. Also, the United Nations Sustainable Development Solutions Network (SDSN) has published *World Happiness Report* annually since 2012, in which the education has also been emphasized as an important factor in people's happiness^2^. Overall, it has almost become a worldwide consensus that education is a key factor in improving people's happiness.

The focus of our investigation is China. As for the largest developing country and the world's second-largest economy by nominal GDP, China has experienced very rapid development over the last four decades. However, the ranking of average happiness level of Chinese people is still relatively low according to the latest *Human Development Report*^3^ and *World Happiness Report* published by United Nations. In the meantime, the gross tertiary school enrollment rate of China has been increasing rapidly since the 1980s, and it has reached roughly 51% in 2018 according to the statistics from the World Bank, which is well above the world average (roughly 38%)^4^. Naturally, a question arises that does higher education is helpful to improve happiness level of Chinese people? In other words, does accumulating human capital mean accumulating happiness in China? Obviously, correctly answering this question is pretty important for both individuals and governments. Because only when the answer of above question is yes, people are more motivated to pursue higher education, and it would make sense for the government to attach more importance to higher education, since Chinese government has proposed to promote people's sense of happiness, many local governments is committed to constructing happy cities, happy societies, and happy communities. Otherwise, there is probably something wrong with the educational systems or the social machines, because happiness is the only ultimate objective for people (Ng and Ho, 2006), higher education should also coincide with the goal of improving happiness.

Using data from four waves of China Household Finance Survey (CHFS), we find that higher education is significantly negatively correlated with people's happiness status. The marginal effect analysis shows that higher education is more likely to prevent people from achieving “extremely happy” lives; instead, it probably lead to “acceptable” lives in urban China. The explanation from housing asset perspective suggests that housing asset plays the mediating role in the relationship between higher education and happiness in China. Specifically, higher education has significantly negative effect on people's years of being homeowner. As a result, higher education would evidently increase the unpaid housing debts and financial costs of housing purchase due to the soaring housing prices in Chinese cities. Meanwhile, higher education has negative effect on people's happiness in cities with relatively high or moderate housing prices, and the higher the housing price is, the lower the happiness of highly educated people would be, but in cities with relatively low housing prices, this effect is insignificant. Moreover, we find that market-oriented housing reform launched in 1998 in urban China has negative impact on highly educated people's happiness. Lastly, Ph.D graduates are the relatively unhappiest people compared to bachelors and masters. We also conduct a series of robustness tests to assure the reliability of our main findings.

Our findings have several important policy implications and marginal contributions. Obviously, it is not a good signal for both individuals and governments that higher education could not lead to happier lives in China. On the one hand, compared to housing wealth accumulation, if human capital investment could reduce people's happiness, it may breed an impetuous social atmosphere and weaken people's motivation to get higher education to some extent. On the other hand, it could further overheat the real estate markets, which would cause more serious social issues and far-reaching social problems. Therefore, Chinese government should continue to take effective measures to suppress housing prices rising too fast and make the real estate market return to rationality and provide some more necessary policy supports for highly educated people to achieve housing dreams.

The rest of the article is structured as follows. Related literature and hypothesis development section reviews the relevant literature on relationship between higher education and people's happiness, discusses potential research gaps, and develops research hypotheses of this article. In Section 3, we introduce our database and discuss the sample and the summary statistics. Section 4 presents the empirical findings in detail, including baseline results, robustness checks, marginal effect analysis, and explanations. We conduct some further discussions in Section 5. Finally, Section 6 provides some concluding remarks and policy implications.

## Related literature and hypothesis development

### Literature review

Overall, the relationship between higher education and people's happiness is still controversy according to the existing literature. Majority of previous studies have found that higher education is positively correlated with people's happiness and wellbeing on regional and worldwide scale. Intuitively, higher education is widely acknowledged as one of the most important investments in human capital, which would provide people many tangible and intangible benefits (Gyimah-Brempong et al., 2006; Winters, 2011; Wang and Liu, 2016; Nikolaev, 2018). Hence, higher education is significant for both satisfaction with life (SWL) and flourishing (Jongbloed, 2018), and it has both direct and indirect effects on one's happiness (Cuñado and Gracia, 2012). In Spain, for instance, higher education could indirectly enhance people's happiness through income and labor status, that is, people with higher education level usually have higher income levels and higher probability of being employed. Also, the “self-confidence” or “self-estimation” effect from acquiring knowledge would have a directly positive impact on happiness (Cuñado and Gracia, 2012). Meanwhile, the empirical evidence from four East Asian countries also confirms that higher education could improve individual's subjective wellbeing *via* enhancing one's ability and propensity to connect with the wider social world, and although both monetary and non-monetary factors play roles in explaining the relationship between higher education and self-reported happiness, monetary factor is relatively unimportant whereas non-monetary factors are important, such as interpersonal network and degree of cosmopolitanism (Chen, 2012). However, China is exceptional because of its relative importance of personal income in accounting for happiness (Chen, 2012). Moreover, some studies find that the extent to which education makes an individual happy depends on their current age in life, highly educated people are more likely to become happier, on average, than their less educated counterparts starting in their early to mid-30s, and beyond this age, the happiness differential starts growing in favor of the more educated (Nikolaev and Rusakov, 2016). In addition, by using longitudinal data from the Household Income and Labor Dynamics in Australia survey, the study shows that people with higher education generally report higher levels of eudaemonic and hedonic subjective wellbeing, and they are satisfied with most life domains (i.e., financial, employment opportunities, neighborhood, local community, and children at home) (Nikolaev, 2018). Also, the positive effect of higher education is increasing, but at a decreasing rate, that is, the happiness gains from obtaining a graduate degree are much lower (on the margin) compared to getting a college degree (Nikolaev, 2018).

However, some economists and educators have come to a totally opposite conclusion. In spite of the obvious economic functionality, school education does not add to personal happiness (Veenhoven, 2010). For example, the empirical evidence from British workers shows that holding income constant, life satisfaction is declining in the level of education, and this may be because education induces higher aspirations that are more difficult to fulfill (Clark and Oswald, 1996). If the happiness is measured as the probability of committing a suicide, it is found that post-secondary education reduces happiness in the United States, since people with college education actually have slightly higher rates of suicide (Buryi and Gilbert, 2014).

Taking together, these previous studies have provided compelling evidence for the relationship between higher education and people's happiness; however, there are still many controversies, and the dimensions of the discussions are still not comprehensive. In particular, there is a lack of in-depth research on the mechanisms behind the relationship, the economic and social environment in different countries are not fully connected, and the research on China are relatively scarce. In other words, the relationship between higher education and people's happiness status in China is still unclear.

### Hypothesis development

Higher education and household's human capital could significantly affect people's housing demand and tenure choice (Logan et al., 2009; Eichholtz and Lindenthal, 2014; Liu and Li, 2018). Specifically, people with higher education usually have more demand for housing (Eichholtz and Lindenthal, 2014), and they (both head and spouse) are more likely to do market purchase, rather than living in market rental, self-built, or collective housings (Logan et al., 2009). However, higher education always means longer time of schooling, which probably further delays the housing purchase. That is, well-educated people are usually latecomers to housing market, although they have much stronger willingness to buy housing units. Since growing literature has found that homeownership and housing wealth accumulation have significantly positive impacts on people's overall happiness (Rossi and Weber, 1996; Hu, 2013; Zumbro, 2014; Tiefenbach and Kohlbacher, 2015; Cheng et al., 2016, 2020; Foye, 2017; Ren et al., 2018; Zhang and Zhang, 2019; Hu and Ye, 2020), in the context of rapid housing appreciation in Chinese cities over the last two decades; one the one hand, these latecomers have to spend more money and borrow more mortgage loans in owning housings. On the other hand, they have missed much economic wealth from housing appreciation. As a result, an unexpected phenomenon would emerge that higher educated people tend to become unhappier. To guide the analysis, we propose hypothesis 1 as follows:

**Hypothesis 1**: Higher educated people tend to postpone housing purchase, and they usually have to spend more money and bear greater financial burdens in owning housings due to the overheated housing market in Chinese cities. Hence, higher education would lead to unhappier life in urban China, which could be partly attributed to the mediating role of housing asset.

Meanwhile, housing prices have major effect on household's tenure choice (Goodman, 1988), which can also affect people's housing affordability and homeowner's housing wealth accumulation, etc. Hence, housing price should be taken into account in explaining people's happiness status. For example, since buying a housing is the largest investment made by most households (Sheiner, 1995), saving rates are responsive to housing prices change, and housing price increases are positively correlated with the savings of young households, suggesting young people are indeed liquidity-constrained (Sheiner, 1995), which would affect their life satisfaction. Also, the average happiness level is positively and significantly related to the change in housing prices for homeowners but not for renters in Canada (Syed, 2016). Similarly, the empirical evidence from Hong Kong shows that the positive correlation between housing price and happiness is valid for older people only, and the rapid rise in the price of housing has made older people happier than youth since the early 2010s (Chiu and Wong, 2018), indicating the young need to bear greater burden in housing costs, since they are mainly renters. Thus, although highly educated people always have better job opportunities and higher income, they are not necessarily very lucky in the housing market due to the soaring housing prices. Considering the huge differentiation in housing prices across Chinese cities (Wu, 2015; Wei et al., 2020), housing assets may have more explanatory power to the life qualities of highly educated people who live in cities with relatively higher housing prices. Accordingly, we propose hypothesis 2 as follows:

**Hypothesis 2**: Higher education has negative effect on people's happiness in cities with relatively high housing prices, while this effect is insignificant in cities with relatively low housing prices.

Furthermore, housing reform in China has proceeded on two tracks, namely privatization of public housing and development of a new private housing sector (Logan et al., 2010). During the era of the planned economy, residential housing was treated as a welfare good rather than a commodity in China, and in the 1980s and until 1998, there was a small-scale program of public housing sales (Chen et al., 2010). In 1998, the market-oriented reform put an end to the welfare allocation of housing, and an increasing proportion of housing is sold directly to the family. Crucially, as the welfare housing provision was terminated in 1998, all new residential housing units built after January 1999 were to be sold on the open market and state-owned enterprises were prohibited from building any more welfare housing for their employees, which finally paved the way for the development of a market-oriented housing sector in urban China (Chen et al., 2010, 2017; Song, 2010). As a result, privatization and commercialization reforms led to the establishment of a commercial housing market, and the shift from centrally planned resource allocation to reliance on markets has fundamentally changed both housing consumption and the macro-economy (Murray and Sun, 2017). However, China's urban housing market has experienced a long-term boom since the housing reform in the 1990s, both transaction volume and housing price have increased rapidly, and even the degree of wealth inequality has been increased substantially due to the rising housing price (Chen et al., 2011, 2017; Li and Wu, 2019). Furthermore, due to lack of talents, the highly educated people were always assigned work units uniformly by Chinese government in last century, and they could get the public housing subsidized prices or even for free. Obviously, housing reform had ended the welfare housing system for these highly educated people, and the value of higher education may be weakened, too. Hence, hypothesis 3 is proposed as follows:

**Hypothesis 3**: The market-oriented housing reform launched in 1998 has negative impact on highly educated people's happiness, since it has essentially changed housing distribution system for urban employees and dramatically boosted housing prices.

## Data

The primary database in this study comes from the China Household Finance Survey (CHFS), which is a nationwide individual-level and household-level data released by Southwestern University of Finance and Economics in China. The CHFS program was conducted *via* face-to-face interviews and standardized questionnaires (Clark et al., 2019). This nationally survey program was started in 2011 and made efforts to collect detailed information mainly about respondent's demographic characteristics, housing assets, finance assets, and subjective attitudes toward life from randomly selected samples every 2 years. Recently, the CHFS database becomes more and more popular in existing studies (Clark et al., 2019; Zhang and Zhang, 2019; Cheng et al., 2020; Huang et al., 2020; Zheng et al., 2020). Basically, there are four waves of available cross-sectional databases from CHFS program so far, which were conducted in 2011, 2013, 2015, and 2017, and the sampling framework totally covers 29 provinces, municipalities, and autonomous regions in mainland China, excluding for Xinjiang and Tibet. To maximize the sample size in our study, we have carefully merged the four waves of CHFS databases at the household level and extracted all the information about the families' heads. Meanwhile, considering that the housing market mainly exists in urban areas, thus we only keep the sample from urban China. Finally, after data cleaning and deletion of missing values, there are approximately 41,797 household observations in our final analytical sample. In addition, the further detailed introductions and questionnaires of CHFS program can be found from its official website^5^.

Importantly, using the CHFS database to carry out this empirical analysis has several clear advantages. First, this database provides rich information about household's housing assets, including the self-assessed total housing value, housing construction space, years of home purchase, and unpaid housing debts. Second, this database contains plenty of information about respondents' educational backgrounds, as well as their parents', which are key independent variables in this study. Third, a lot of other necessary variables for our empirical process are also contained in the CHFS questionnaires, including household demographic characteristics (i.e., gender, age, marital status, family size, and health status), socioeconomic characteristics (i.e., *hukou* status, political identity, home ownership, disposable income, total debts, car ownership, medical insurance participation, and pension insurance participation), since they are verified to be critical in explaining people's happiness status according to related studies.

Besides the micro-level variables picked up from the CHFS database, considering that there are several macro-level variables that may have impacts on people's subjective wellbeing, such as geographical location, house prices, regional economic development, and local population scale. Therefore, to control for these variables in our empirical models, we collect the data of housing prices, GDP per capita, and population scale from China Statistical Yearbooks, which are published by the National Statistics Bureau of China every year^6^. In addition, we also classify the geographical location for each sample based on the regulation from the National Statistics Bureau of China, the region of eastern China contains 12 provinces, municipalities, and autonomous regions, namely Beijing, Tianjin, Hebei, Liaoning, Shanghai, Jiangsu, Zhejiang, Fujian, Shandong, Guangdong, Guangxi, and Hainan.

**Figure 1:**
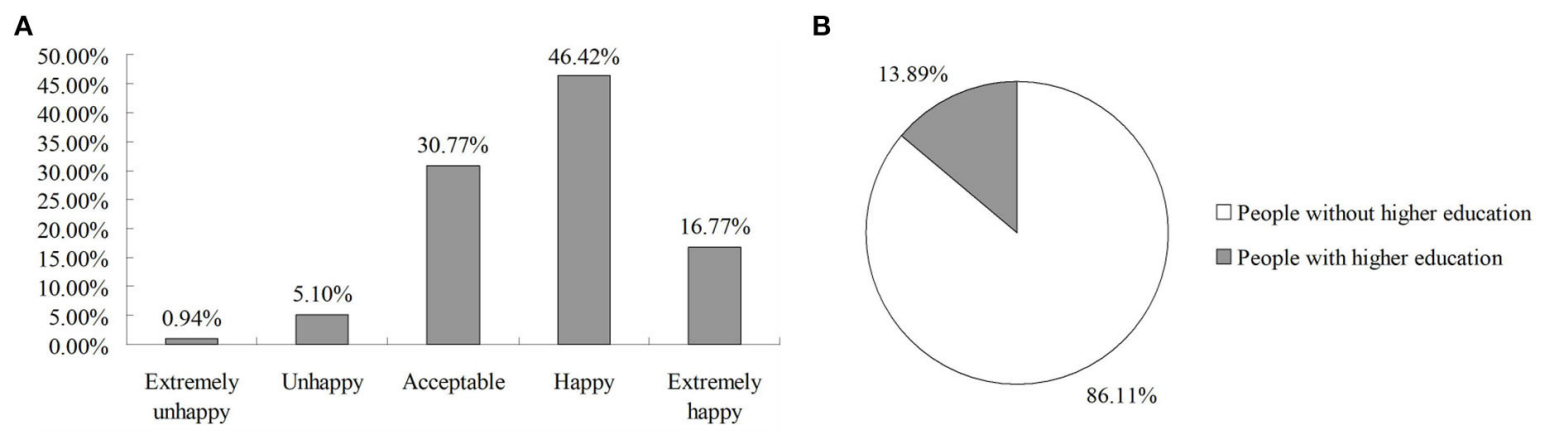
Preliminary statistics. (1) *Data source*: 2011, 2013, 2015, and 2017 waves of CHFS. (2) The self-reported happiness with current life is measured on a five-point scale, ranging from “extremely unhappy” to “extremely happy.” (3) The higher education in this study is defined as an indicator variable, and people with higher education means they have got bachelor degree, master degree, or Ph.D degree when the survey was conducted. **(A)** frequency distribution of happiness. **(B)** percentage of higher educated people.

Based on our final analytical sample, Figure 1 illustrates the frequency distribution of people's happiness status and percentage of higher educated people. As for the happiness distribution, which is measured on a five-point scale, ranging from “extremely unhappy” to “extremely happy.” Overall, there are roughly 46.42% people feel “happy” in their lives in urban China, which accounts for the largest proportion compared to other happiness statuses. Also, around 16.77% people highly evaluate their lives, and they feel “extremely happy.” Hence, what we can conclude that more than 60% people in urban China are experiencing happy lives or happiest lives. Besides, about 30.77% people think their lives are “acceptable,” and there are still a few people (roughly 6%) feel “unhappy” or even “extremely unhappy” in their lives. Meanwhile, in terms of the percentage of higher educated people, which are defined as these people who have got bachelor degree, master degree, or Ph.D degree when they are interviewed. As shown in Figure 1, there are around 13.89% heads of households have been higher educated, while the remaining respondents (roughly 86.11%) have not experienced higher education. According to some related studies, this proportion is still evidently lower than that in advanced economies, but the university enrollment rate in China is increasing rapidly in recent years.

Table 1 reports the description and summary statistics for each explained variable, explanatory variable, and other control variables in this study. Most of these variables are categorical, either qualitative or binary. Specifically, the interviewees in our sample are selected among citizens aged above 18 at random, and their average age is about 51 years old. About 69% heads of households are men, 65% respondents hold urban *hukou* identity, and approximately half of full sample come from eastern China; accordingly, the other half of sample are from central, northeast, or western China; this is mainly because that much more population distributes in eastern China. Overall, this micro-level database from CHFS is well represented for urban China.

**Table 1:** Variable description and summary statistics.

**Variable**	**Descriptions**	**Mean**	**Std. Dev**.	**Min**	**Max**
Happiness status	An ordered variable of self-reported happiness with current life, which is measured on a five-point scale, ranging from 1 (Extremely unhappy) to 5 (Extremely happy)	3.731	0.834	1	5
Higher education	A binary variable that equals to one if the respondent has got bachelor degree, master degree or Ph.D degree, and equals to zero otherwise	0.139	0.346	0	1
Father_Higher education	A binary variable that equals to one if the respondent's father has got bachelor degree, master degree or Ph.D degree, and equals to zero otherwise	0.018	0.131	0	1
Mother_Higher education	A binary variable that equals to one if the respondent's mother has got bachelor degree, master degree or Ph.D degree, and equals to zero otherwise	0.017	0.128	0	1
Urban *hukou*	A binary variable that equals to one if the respondent holds non-agricultural *hukou* registration, and equals to zero otherwise	0.650	0.477	0	1
Gender	A binary variable that equals to one if the respondent is male, and equals to zero if the respondent is female	0.690	0.462	0	1
Age	The age of the head of household when the survey conducted	50.994	15.601	18	100
Unmarried	A binary variable of respondent being single	0.066	0.249	0	1
Married	A binary variable of respondent being married	0.824	0.381	0	1
Divorced	A binary variable of respondent being divorced	0.030	0.170	0	1
Family size	Total number of family members living together	2.459	1.534	0	15
Only child	A binary variable of people without any brothers or sisters	0.363	0.481	0	1
Health status	An ordered variable of self-reported health status measured on a five-point scale and evaluated by respondent, ranging from 1 (extremely unhealthy) to 5 (extremely healthy)	3.237	1.098	1	5
Education	The education years for the respondent to get the highest degree	15.878	5.269	0	22
Employed	A binary variable of people being employed	0.586	0.493	0	1
communist	A binary of respondent being a member of Communist party of China	0.190	0.392	0	1
Working hour	The average working hours per day (*hours*)	8.523	2.409	0	18
homeownership	A binary variable that equals to one if the respondent has already owned his/her house, and equals to zero otherwise	0.854	0.353	0	1
Income	Total household disposable income in the last year (*yuan*)	93,545.620	198,260.300	0	5,000,000
Debt	Total amount of household's unpaid debts (*yuan*)	59,963.010	904,057.000	0	10,000,000
Car	A binary variable of people owning a car	0.257	0.437	0	1
Medical insurance	A binary variable that equals to one if the respondent has joined in the urban essential medical insurance or other public health services, and equals to zero otherwise	0.894	0.307	0	1
Pension insurance	A binary variable that equals to one if the respondent has joined in the urban essential pension insurance or other public pension services, and equals to zero otherwise	0.793	0.405	0	1
Eastern China	A binary variable that equals to one if the sample comes from eastern China	0.501	0.500	0	1
Housing price	Average sales price of commercial housing at provincial level (*yuan per square meter*)	8,434.105	5,749.445	3,629	34,117
Population	The total regional population of permanent residents at the end of the year (*10 thousand*)	5,217.978	2,975.524	578	11,169
GDP per capita	Regional gross domestic product per capita (*yuan*).	62,185.390	26,151.960	23,151	12,8994
Urban CPI	The index of the urban consumer price index	102.029	0.684	100.6	104.1

Data source: 2011, 2013, 2015, and 2017 waves of CHFS (Available at: https://chfser.swufe.edu.cn/datasso/Home/Login); China's National Bureau of Statistics (Available at: http://www.stats.gov.cn/).

## Empirical findings

In this section, we make efforts to investigate the relationship between higher education and people's happiness status in detail and try to give some plausible explanations from housing asset perspective. Specifically, we first present the evidence that higher education in urban China is negatively correlated with people's overall happiness, and based on the robustness checks and marginal analysis, we then explain this seemingly abnormal phenomenon from the perspective of individual housing asset, including years of being homeowner, unpaid housing debt, financial cost of housing purchase, and regional housing prices. Moreover, giving the fact that housing reform launched in 1998 in urban China had fundamentally changed housing market, as well as housing distribution system for urban employees, we further examine the impact of housing reform on the relationship between higher education and people's happiness status.

### Benchmark result

To study the relationship between higher education and personal happiness status, and considering that the happiness status in CHFS survey is measured on a five-point scale ranging from 1 (extremely unhappy) to 5 (extremely happy), thus we employ ordered probit model to conduct the empirical process. The estimation function is structured as the following form:


(1) $$\begin{array}{l} Happiness_{it}=\alpha_{0,1}+\alpha_{0,1}Higher\;education_{it}+\alpha_{1,1}X_{it}\\ \;+\vartheta_{j}+\sigma_{t}+\mu_{it,1} \end{array}$$

where subscript *i* denotes the individual and *t* is the specific wave of the CHFS survey. *Happiness*_*it*_ is the dependent variable that represents respondent's self-reported happiness status. *Higher education*_*it*_ is an indicator variable for these respondents who have obtained bachelor degree, master degree, or Ph.D degree, which is also the independent variable in this study. *X*_*it*_ is a vector of all control variables, including household demographic characteristics (i.e., gender, age, marital status, family size, only child or not, and health status), socioeconomic characteristics (i.e., employment, political identity, working hours, homeownership, *hukou* registration, disposable income, household debts, car ownership, medical insurance, and pension insurance), and regional level variables (i.e., geolocation, housing price, population scale, GDP per capita, and urban CPI). Also, since our data cover 29 provinces, municipalities, and autonomous regions in mainland China and comprise four waves of CHFS surveys, we further control for regional fixed effect (ϑ_*j*_) and year fixed effect (σ_*t*_) in our empirical model. In addition, μ_*it*, 1_ is the error term.

We estimate a series of different specifications by gradually increasing the number of control variables into vector *X*_*it*_, so as to see their effects on respondent's happiness status change. The estimated coefficients, robust clustered standard errors, and significance levels of the independent variable and control variables are reported in Table 2, which is also the benchmark result in our study and presents the relationship between higher education and people's overall happiness in urban China.

**Table 2:** Relationship between higher education and people's happiness status.

	**(1)**	**(2)**	**(3)**	**(4)**
	**Dependent variable: Happiness status (OPM)**			
**Independent variable**				
Higher education	0.088^***^	0.042^*^	−0.088^***^	−0.088^***^
	(0.028)	(0.023)	(0.022)	(0.022)
**Control variable**				
Demographic characteristics				
Gender		−0.094^***^	−0.101^***^	−0.102^***^
		(0.016)	(0.014)	(0.014)
Age		−0.038^***^	−0.043^***^	−0.043^***^
		(0.002)	(0.002)	(0.002)
(Age∧2)/1,000		0.431^***^	0.460^***^	0.460^***^
		(0.023)	(0.025)	(0.025)
Unmarried		−0.219^***^	−0.214^***^	−0.213^***^
		(0.040)	(0.039)	(0.038)
Married		0.273^***^	0.224^***^	0.224^***^
		(0.021)	(0.021)	(0.021)
Divorced		−0.135^***^	−0.127^***^	−0.127^***^
		(0.019)	(0.020)	(0.020)
Family size		0.000	−0.013^**^	−0.013^**^
		(0.005)	(0.005)	(0.005)
Only child		0.060^***^	0.062^***^	0.063^***^
		(0.018)	(0.020)	(0.020)
Health status		0.272^***^	0.254^***^	0.254^***^
		(0.008)	(0.008)	(0.008)
Socioeconomic characteristics				
Employed			0.059^**^	0.059^**^
			(0.024)	(0.024)
Communist			0.116^***^	0.116^***^
			(0.013)	(0.013)
Working hours			−0.008^***^	−0.008^***^
			(0.003)	(0.003)
Homeownership			0.151^***^	0.151^***^
			(0.023)	(0.022)
Urban hukou			−0.028	−0.028
			(0.019)	(0.019)
Ln (Income)			−0.043^***^	−0.043^***^
			(0.007)	(0.007)
Ln (Income)∧2			0.005^***^	0.005^***^
			(0.000)	(0.000)
Ln (Debt)			−0.013^***^	−0.013^***^
			(0.001)	(0.001)
Car			0.166^***^	0.166^***^
			(0.016)	(0.016)
Medical insurance			0.048^***^	0.049^***^
			(0.017)	(0.017)
Pension insurance			0.066^***^	0.065^***^
			(0.020)	(0.020)
Regional level variables				
Eastern region				−0.826
				(0.624)
Ln (Housing price)				−0.040
				(0.123)
Ln (Population)				0.715
				(0.497)
Ln (GDP per capita)				0.091
				(0.078)
Urban CPI				0.016
				(0.026)
Intercept	0.960^***^	1.342^***^	1.383^***^	8.333^**^
	(0.020)	(0.073)	(0.080)	(3.668)
Year dummies	Yes	Yes	Yes	Yes
Region dummies	Yes	Yes	Yes	Yes
Observations	41,797	41,797	41,797	41,797

(1) Robust and clustered standard errors in parentheses; (2)

* *p* < 0.1,

** *p* < 0.05, and

*** *p* < 0.01.

Specifically, we begin with the simplest specification *via* only controlling for higher education, as well as the year and regional dummies, and the regression result is reported in column (1) of Table 2. The coefficient is positive and it is significant at the 1% level, which means that without controlling for any other observable factors, people who have been highly educated are averagely much happier than others. We then add the demographic characteristics to the model, including gender, age, age square term, marital status, family size, only child or not, and self-assessed health status. The result is shown in column (2) of Table 2. In this specification, the coefficient of higher education still remains positive but the gap is merely significant at the 10% level, suggesting that the relationship between higher education and people's happiness is changing rapidly after controlling for some individual-level variables. Furthermore, we continue to control for respondent's socioeconomic characteristics, including employment, political identity, working hours, homeownership, *hukou* registration, disposable income and its square term, household debts amount, medical insurance, and pension insurance participation. As shown in column 3 of Table 2, the coefficient of higher education becomes negative and the difference is statistically significant at the 1% level. That is, the sign of the independent variable is totally reversed after controlling for the socioeconomic characteristics, which means the higher education generally has negative effect on people's happiness status. Lastly, as reported in column 4, several regional level variables are controlled for, such as geolocation, local housing price, population scale, GDP per capita, and urban CPI, the regression result is in line with that in column 3. Hence, we can basically come to a conclusion that higher education is negatively correlated with people's self-reported happiness in urban China. However, compared to the results based on another countries' empirical evidence, this basic finding seems to against the recent findings in some existing literature (Smyth et al., 2010; Cuñado and Gracia, 2012; Nikolaev and Rusakov, 2016), although some related researches have put forward similar conclusion (Veenhoven, 1996; Hartog and Oosterbeek, 1998).

### Robustness check

In this section, we aim to address the potential issues behind the baseline results in previous Table 2 and try our best to make sure the basic finding in this study is robust and convincing. Besides the estimation approach, we pay more attention on the possible endogenous issues and model misspecification. Therefore, the robustness checks are mainly conducted *via* different estimation approaches (i.e., ordered logit model, OLS model, and probit-adapted OLS approach), propensity score matching approach (PSM), and instrumental variable approach (IV).

#### Re-estimated by OLM and OLS

Given that the happiness status in CHFS survey is measured by a five-point scale and ranging from 1 (extremely unhappy) to 5 (extremely happy), besides the ordered probit model (OPM) that we have employed in Table 2, the ordered logit model (OLM) is also a widely used method for ordinal dependent variables. In the meantime, there are also a bunch of existing studies that use the traditional ordinary least square (OLS) model to estimate the regression equations with ordinal dependent variables (Jiang et al., 2012; Hu and Ye, 2020; Zheng et al., 2020). Hence, we re-estimate the Equation (1) through OLM and OLS approaches.

As reported in Table 3, the regression results in columns (1) and (2) are re-estimated by OLM and OLS approaches, respectively. The results show that the coefficients re-estimated by these two approaches are also negative and they are all statistically significant at the 1% level, which are consistent with the earlier baseline result.

**Table 3:** Robustness checks.

	**(1)**	**(2)**	**(3)**	**(4)**	**(5)**
	**OLM**	**OLS**	**PSM**		
			**Nearest neighbor matching**	**Kernel matching**	**Mahalanobis matching**
**Independent variable**				
Higher education	−0.152^***^	−0.062^***^	−0.067^***^	−0.090^***^	−0.071^***^
	(0.039)	(0.016)	(0.024)	(0.022)	(0.026)
**Control variable**				
Demographic characteristics	Yes	Yes	Yes	Yes	Yes
Socioeconomic characteristics	Yes	Yes	Yes	Yes	Yes
Regional level variables	Yes	Yes	Yes	Yes	Yes
Year dummies	Yes	Yes	Yes	Yes	Yes
Region dummies	Yes	Yes	Yes	Yes	Yes
Intercept	Yes	Yes	Yes	Yes	Yes
Observations	417,97	41,797	16,035	41,346	16453

(1) Robust and clustered standard errors in parentheses; (2)

*** *p* < 0.01.

#### Propensity score matching approach

Furthermore, our previous estimations are based on linear impacts of covariates on the outcome variable. If their relationship is non-linear, our previous estimations may be biased due to functional misspecification. To deal with this potential issue, we apply the propensity score matching (PSM) approach to re-generate a new sampling framework, which is pretty popular to create a balanced covariate distribution between treated and untreated groups. Specifically, we use three different matching algorithms to match the higher educated group (treatment group) and the other group (comparison group), including the nearest neighbor matching, Kernel matching, and Mahalanobis matching. Indeed, before conducting the PSM estimation, we have checked the matching qualities to make sure the estimations of previous models are valid, and the matching procedures balance the distribution of the controlling variables in both treatment groups and comparison groups. Also, the common support conditions of the PSM are checked as well, which has assured there is common support of the propensity score distributions of the two groups.

The PSM estimation results based on three approaches are reported in columns (3)–(5) of Table 3. Clearly, the coefficients of interest are negative and statistically significant at the 1% level, which are highly consistent with the baseline result in this study. Hence, the PSM estimation results one again prove that higher education is negatively correlated with people's happiness status in urban China.

#### Instrumental variables estimation

To address the possible issue of endogeneity, that is, the explanatory variable of interest may be correlated with the error term in our regression model. Generally, as for the causal effect estimation, one of the widely used methods to handle this potential issue is using instrumental variables approach (IV), which allows for consistent estimation in theory. Meanwhile, according to series of existing literature, parents' educational levels are often regarded as the instrumental variable of individual's schooling (Pons and Gonzalo, 2002; Lee and Fish, 2010; Bhatti et al., 2013). Thus, we also employ individual's parents' educational levels (i.e., father's educational level and mother's educational level) as IVs to conduct two-stage least squares (2SLS) regression analysis, and the 2SLS analysis comprises IV-OLS and IV-OPM.

The results of IV estimation are displayed in Table 4, where we have reported the results of the first stage and the second stage, as well as the F statistics for the first stage. Specifically, after using father's educational level and mother's educational level as IVs, the coefficients in columns (2) and (4) are both negative and statistically significant at the 5% level, which means the higher education is negatively correlated with people's happiness status. Obviously, the results estimated by IV-OLS are in line with the baseline. Similarly, the coefficients in columns (6) and (8) are significantly negative at the 1% level, which again suggests the benchmark result in our study is robust.

**Table 4:** Results of IV estimation.

	**(1)**	**(2)**	**(3)**	**(4)**	**(5)**	**(6)**	**(7)**	**(8)**
	**IV-OLS**				**IV-OPM**			
	**First stage**	**Second stage**	**First stage**	**Second stage**	**First stage**	**Second stage**	**First stage**	**Second stage**
**Independent variable**								
Higher education		−0.362^**^		−0.290^**^		−0.332^***^		−0.273^***^
		(0.147)		(0.121)		(0.125)		(0.104)
**Instrumental variable**								
Father_Higher education	0.200^***^				0.325^***^			
	(0.015)				(0.013)			
Mother_Higher education			0.240^***^				0.402^***^	
			(0.016)				(0.013)	
**Control variable**								
Demographic characteristics	Yes	Yes	Yes	Yes	Yes	Yes	Yes	Yes
Socioeconomic characteristics	Yes	Yes	Yes	Yes	Yes	Yes	Yes	Yes
Regional level variables	Yes	Yes	Yes	Yes	Yes	Yes	Yes	Yes
Year dummies	Yes	Yes	Yes	Yes	Yes	Yes	Yes	Yes
Region dummies	Yes	Yes	Yes	Yes	Yes	Yes	Yes	Yes
Intercept	Yes	Yes	Yes	Yes	Yes	Yes	Yes	Yes
F statistics	175.36	—	177.42	—	645.19	—	937.99	—
Observations	41,797	41,797	41,797	41,797	41,797	41,797	41,797	41,797

(1) Robust and clustered standard errors in parentheses; (2)

** *p* < 0.05, and

*** *p* < 0.01.

#### Re-estimated by probit-adapted OLS approach

Furthermore, in terms of the dependent variable in our study, instead of arbitrarily assigning values 1, 2, 3, 4, or 5 to the five possible answers to the happiness question, we employ the probit-adapted OLS (or probit-OLS, POLS) method to assign these values (van Praag and Ferrer-i-Carbonell, 2008). This method consists of assigning values to match the distribution of responses to a normal distribution. For example, if a fraction *q* reports the lowest category (“extremely unhappy”), the probit-adapted OLS method assigns the lowest category a score of E[z|z<q], where *z* is distributed standard normal. Recently, Perez-Truglia (2020) has used this method to study the effectiveness of income transparency on people's wellbeing. Hence, we use the probit-adapted OLS method to further conduct the robustness test, and the resulting values for the happiness scores are 1.606 (“extremely happy”), 0.292 (“happy”), −0.90 (“acceptable”), −1.99 (“unhappy”), and −2.88 (“extremely unhappy”). The result re-estimated by probit-adapted OLS approach is reported in Table 5. Clearly, it also highly consistent with the result listed in previous Table 2, again suggesting the benchmark result in our study is robust.

**Table 5:** Re–estimated by probit–adapted OLS approach.

	**(1)**	**(2)**	**(3)**	**(4)**
	**Dependent variable:**			
	**Happiness status (Probit–adapted OLS)**			
**Independent variable**				
Higher education	0.081^***^	0.037^*^	−0.077^***^	−0.076^***^
	(0.026)	(0.020)	(0.019)	(0.019)
**Control variable**				
Demographic characteristics	No	Yes	Yes	Yes
Socioeconomic characteristics	No	No	Yes	Yes
Regional level variables	No	No	No	Yes
Intercept	Yes	Yes	Yes	Yes
Year dummies	Yes	Yes	Yes	Yes
Region dummies	Yes	Yes	Yes	Yes
Observations	41,797	41,797	41,797	41,797
adjusted R^2^	0.018	0.094	0.110	0.110

(1) Robust and clustered standard errors in parentheses; (2)

* *p* < 0.1 and

*** *p* < 0.01.

### Marginal analysis

Considering that the meaning of coefficient estimated by ordered probit model is not intuitive, in terms of the baseline result in previous Table 2 in this study, we can only extract limited information from coefficient's sign and its significance level. However, we are also curious about the marginal effect of higher education on people's overall happiness, since we would obtain more valuable understandings their relationship through comparison of marginal effects. Specifically, we could further answer the question that what are the different impacts of higher education on various happiness status in China *via* marginal analysis.

Therefore, we would like to calculate how the unit change of explanatory variable and how the influence the probability of the explained variable taking various values when all other control variables are at the mean. The formula takes the following form:


(2) $$ME(Happiness\;status)={\frac{\partial prob(y=n|x)}{\partial (x)}}_{{|}_{x=\bar{x}}}(n=1,2,3,4,5)$$

where *x* and *y* represent independent variable and dependent variable in Formula (2), respectively, and *n* stands for a set of possible values for *y*. As reported in Table 6, when all other control variable are at the mean, higher education has significantly positive effects on the probabilities of “extremely unhappy,” “unhappy,” and “acceptable,” while it has significantly negative impacts on the probabilities of “happy” and “extremely happy.” In other words, higher education could evidently lower people's happiness status, which is in line with the baseline results. Furthermore, higher education has relatively tiny effects on the probabilities of “extremely unhappy” and “unhappy,” while it could affect the probabilities of “acceptable,” “happy,” and “extremely happy” to a greater extent. In particular, higher education could generally lower the probabilities of “happy” and “extremely happy” by 0.0104 and 0.0208, respectively. That is, higher education is more likely to prevent people being extremely happy. Obviously, this is the unexpected outcome that higher education and happiness status run counter to each other in China.

**Table 6:** Marginal effect of higher education on overall happiness.

**Happiness status**	**Marginal effect (*Higher education*)**	**Delta–method** **standard error**	**Z statistics**
Extremely unhappy	0.0020^***^	0.001	3.98
Unhappy	0.0078^***^	0.002	4.07
Acceptable	0.0213^***^	0.005	3.93
Happy	−0.0104^***^	0.003	−3.85
Extremely happy	−0.0208^***^	0.005	−4.04

*** *p* < 0.01.

### Possible explanations

Naturally, a question arises that what are the possible reasons for the negative relationship between higher education and people's happiness status in urban China? Some of existing literature has argued that this kind of negative relationship does not mean education itself breeds dissatisfaction; instead, the dissatisfaction among the highly educated is probably due to a lack of jobs at that level and possibly to the fading of earlier advantages in the process of social equalizing (Veenhoven, 1996). Meanwhile, the highest level of education neither produces the highest wealth, nor the highest health nor the highest happiness, and the parabolic effect of schooling on happiness is mostly created through the parabolic effect on health and wealth (Hartog and Oosterbeek, 1998). Inspired by these existing research results, we would like to explore the possible explanation for the basic finding in this study from the perspective of household's housing assets, since there is a bunch of existing literature which have found that housing is a key element affecting the quality of human life because it can fulfill several human needs (Chiu and Wong, 2018), and homeownership and housing wealth accumulation have significantly positive impact on people's overall happiness (Hu, 2013; Cheng et al., 2016, 2020; Foye, 2017; Ren et al., 2018; Zhang and Zhang, 2019; Hu and Ye, 2020).

#### Years of schooling vs. years of being homeowner

Notably, the soaring housing prices are one of the most prominent characteristics of Chinese cities over the last two decades (Chen et al., 2019). According to the official data released by China's National Bureau of Statistics, the housing price in urban China has experienced continuously rapid rising in the new century, which can be largely attributed to the housing reform that launched in 1998. The housing market-oriented reform completely ended the welfare housing system and opened a new era for housing market in urban China. As a result, the annual growth rate of housing prices often surpassed 10% or even 20%.

Although the fluctuation of the housing prices growth rate has narrowed somewhat after 2010, it still has been at a high level. In other words, people who obtain their homeownership earlier always means they could get more economic benefits from housing appreciation, which is usually summarized as housing wealth effect (Kishor, 2007; Peltonen et al., 2012; Cooper, 2013; Khalifa et al., 2013; Liao et al., 2014).

Given that the years of being homeowner could significantly affect household's housing wealth in urban China, does the years of schooling shorten the years of being homeowner? If it does, the negative relationship between higher education and people's happiness status could be partly explained by housing wealth accumulation. Hence, we continue to conduct the empirical analysis of the relationship between higher education and years of being homeowner. The estimation model is structured as follows:


(3) $$\begin{array}{l} Year\_homeowner_{it}=\alpha_{0,2}+\alpha_{1,2}Higher\;education_{it}\\ \;+\alpha_{2,2}X_{it}+\vartheta_{j}+\sigma_{t}+\mu_{it,2} \end{array}$$

where *Year*_*homeowner*_*it*_ stands for the years of being homeowner of the household head, *Higher education*_*it*_ is still an indicator variable for these respondents who have been highly educated, and the other variables in Equation (3) are the same as those in previous Equation (1). After excluding the samples that lack the years of being homeowner, there are roughly 29,968 household observations in our final analytical sample. We then employ the traditional ordinary least squares (OLS) method to estimate Equation (3).

As indicated in Table 7, the controls are gradually added into the models, and the coefficients from columns (1)–(4) are all negative and they are statistically significant at the 1% level. That means higher education could evidently compress the years of being homeowner. In other words, holding everything is equal, the time of highly educated people buying homes are generally much later than those who have not entered universities. Therefore, against the backdrop of soaring housing prices in urban China, highly educated people are more likely to miss the “good opportunity” of purchasing housings, and the opportunity cost makes them benefit less from housing appreciation. Indeed, it would evidently lower people's happiness status (Clapham, 2010; Zhang and Zhang, 2019).

**Table 7:** Relationship between higher education and years of being homeowner.

	**(1)**	**(2)**	**(3)**	**(4)**
	**Dependent variable:**			
	**Years of being homeowner**			
**Independent variable**				
Higher education	−3.821^***^	−1.648^***^	−0.921^***^	−0.916^***^
	(0.232)	(0.190)	(0.181)	(0.182)
**Control variable**				
Demographic characteristics	No	Yes	Yes	Yes
Socioeconomic characteristics	No	No	Yes	Yes
Regional level variables	No	No	No	Yes
Year dummies	Yes	Yes	Yes	Yes
Region dummies	Yes	Yes	Yes	Yes
Intercept	Yes	Yes	Yes	Yes
Observations	29,968	29,968	29,968	29,968

(1) Robust and clustered standard errors in parentheses; (2)

*** *p* < 0.01.

Furthermore, higher education always means longer time of schooling, but it does not necessarily mean that people cannot buy housings while studying at universities. Thus, a question arises naturally that why people do not buy homes before they graduate from universities? Indeed, we cannot deny the fact that some people have already become homeowners when they were still students because of the inter-generational support, suggesting that they have not only achieved human capital accumulation, but also accumulated housing wealth. However, higher education does averagely compress the years of being homeowner according to the result in Table 6. Based on some realities in urban China, we can summarize the main reasons for above question as follows.

First, higher education is actually a type of human capital investment, which would cost pretty much money to pay for tuition and living expenses, and some relatively poor people even have to borrow money or loans from their relatives or banks. Thus, the education investment will largely consume the money invested in housing to a great extent, and they have no enough money to purchase homes due to the limited affordability. Second, unlike the people who are hired, university students generally do not have stable income in China, even if they attempt to buy homes and realize housing appreciation, they lack the abilities to repay the housing mortgage loans. In principle, banks do not lend housing loans to students as they have no stable jobs and cash flow, so the schooling students have to postpone the time of being homeowner. Third, in addition to lack of financial availability, higher education often delays people's employment and marriage time (Cherlin, 1980; Wan, 2006), which also puts off or weaken the motivation of buying homes for these highly educated people. Last but not least, although higher education normally leads to higher income and social status, the return on housing investment is even higher than the return on higher education due to the housing price soaring, and highly educated people also need to face the big pressure in owning housings.

#### The financial cost of buying homes

Since the years of schooling could significantly compress people's years of being homeowner according to the findings in Table 7, and considering the rapid rising in housing prices in urban China, it is reasonable to draw the inference that higher education would lead to higher financial costs of housing purchase. Therefore, we are going to empirically prove this inference, and in this study, the housing financial costs consist of unpaid housing debt and the cost of per square meter housing. We thus conduct the following regression:


(4) $$\begin{array}{l} Cost_{it}=\alpha_{0,3}+\alpha_{1,3}Higher\;education_{it}+\alpha_{2,3}X_{it}+\vartheta_{j}\\ \;+\sigma_{t}+\mu_{it,3} \end{array}$$

where *Cost*_*it*_ represents the housing financial costs, and the other variables in Equation (4) are the same as those in Equation (1).

First, we investigate the relationship between higher education and unpaid housing debt, and the unpaid housing debt is measured in two ways, including whether has unpaid housing debt and the amount of unpaid housing debt. Obviously, the former one is an indicator variable while the later one is a continuous variable. Therefore, we use probit and OLS approach to estimate Equation (4), respectively.

As shown in Table 8, columns (1) and (2) report the empirical result when the dependent variable is in its binary form. The coefficients are positive, and they are statistically significant at the 1% level regardless of whether the demographic characteristics, socioeconomic characteristics, and regional level variables are controlled for, suggesting that highly educated people are more likely to still have outstanding housing debt. In the meantime, columns (3) and (4) are the other scenario where the dependent variable is the amount of unpaid housing debt. Consistently, the coefficients are also significantly positive. Specifically, the coefficient is 0.977 in column (4), which indicates that the housing debt of people with higher education is on average 97.7% higher than others. The almost doubled housing debt means welleducated people are currently under greater financial pressure on housing, and the reason can be summed up in two points. On the one hand, highly educated people generally buy homes much later than others according to earlier finding in this study, so they have more remaining housing debt. On the other hand, due to the soaring housing prices, people who purchase housing late usually need to borrow more mortgage loans from banks. Considering the household debt would significantly lower people's happiness (Tay et al., 2017; Liu et al., 2020), the greater pressure come from more housing debt would make highly educated people unsatisfied with their lives.

**Table 8:** Relationship between higher education and unpaid debt.

	**(1)**	**(2)**	**(3)**	**(4)**
	**Dependent variable:**		**Dependent variable:**	
	**whether has unpaid housing debt**		**Logarithm of amount of unpaid housing debt**	
	**Probit**		**OLS**	
**Independent variable**				
Higher education	0.635^***^	0.337^***^	1.603^***^	0.977^***^
	(0.029)	(0.021)	(0.111)	(0.077)
**Control variable**				
Demographic characteristics	No	Yes	No	Yes
Socioeconomic characteristics	No	Yes	No	Yes
Regional level variables	No	Yes	No	Yes
Year dummies	Yes	Yes	Yes	Yes
Region dummies	Yes	Yes	Yes	Yes
Intercept	Yes	Yes	Yes	Yes
Observations	41,797	41,797	41,797	41,797

(1) Robust and clustered standard errors in parentheses; (2)

*** *p* < 0.01.

Then, we move to test the relationship between higher education and the unit costs of owning homes, and there are around 32,158 valid observations after excluding the sample with missing values of housing unit cost. As suggested in Table 9, we gradually increase the control variables into estimation models, the coefficients keep positive, and the differences are statistically significant at the 1% level. In particular, when all the observables are controlled for in column (4), the coefficient of interest is 0.461, indicating that the unit cost of owning homes of highly educated people is generally 46.1% higher than others. This finding once again confirms that higher education leads to an increase in the cost of buying housing, and one of the most important reasons is that highly educated people are more likely to miss the time window for buying a home early when the housing prices are relatively low. Hence, as for those highly educated people, greater pressure in buying housing will reduce their happiness. In summary, the hypothesis 1 has been well confirmed based on the above empirical findings.

**Table 9:** Relationship between higher education and the unit cost of owning homes.

	**(1)**	**(2)**	**(3)**	**(4)**
	**Dependent variable:**			
	**Logarithm of unit cost of owning**			
	**homes (per square meter)**			
**Independent variable**				
Higher education	1.119^***^	0.821^***^	0.461^***^	0.461^***^
	(0.059)	(0.061)	(0.043)	(0.043)
**Control variable**				
Demographic characteristics	No	Yes	Yes	Yes
Socioeconomic characteristics	No	No	Yes	Yes
Regional level variables	No	No	No	Yes
Year dummies	Yes	Yes	Yes	Yes
Region dummies	Yes	Yes	Yes	Yes
Intercept	Yes	Yes	Yes	Yes
Observations	32,158	32,158	32,158	32,158

(1) Robust and clustered standard errors in parentheses; (2)

*** *p* < 0.01.

#### Difference caused by regional housing prices

Considering that the soaring housing prices play a vital role in the relationship of higher education and people's happiness status according to the earlier analysis, and it is a confirmed fact that there is obvious differentiation in housing prices across Chinese cities (Wu, 2015; Wei et al., 2020). Therefore, it is necessary to take the regional housing prices into account. Specifically, we would like to classify all the 41,797 observations into three subgroups by the tertile of regional housing prices, and the regional housing prices at the provincial level are collected from *China's National Statistical Yearbook*, which is published by National Bureau of Statistics once a year. As a result, these three subgroups marked as regions with relatively high housing price, moderate housing price, low housing price include 12,446, 16,360, and 12,991 samples, respectively.

Table 10 reports the impacts of higher education on people's happiness among regions with different housing prices. The coefficient in column (1) is negative and it is statistically significant at the 1% level, suggesting higher education is negatively correlated with people's happiness in these regions with relatively high housing price. Similarly, the result in column (2) shows that highly educated people also tend to be unhappy in regions with relatively moderate housing price, and the gap is statistically significant at the 5% level. However, in terms of the regions with relatively low housing price, as shown in column (3), the estimation coefficient of higher education and people's happiness status is negative but insignificant. In other words, the highly educated people who live in cities with higher housing prices are more likely to be unhappy or unsatisfied with their lives. Moreover, we also retest above finding by using interaction between housing price and higher education in column (4), the coefficient of higher education becomes positive at the 10% level, but the log of regional housing price has significantly negative impact on people's happiness status, and the sign of interaction is also significantly negative. Again, this finding provides evidence that the higher the housing prices, the lower the happiness of highly educated people. Therefore, the hypothesis 2 is empirically supported.

**Table 10:** The impacts of higher education on people's happiness among different regions.

	**(1)**	**(2)**	**(3)**	**(4)**
	**Dependent variable: Happiness status (OPM)**			
	**Subgroup 1**	**Subgroup 2**	**Subgroup 3**	**Full sample**
	**Regions with relatively high housing price**	**Regions with relatively moderate housing price**	**Regions with relatively low housing price**	**Interaction**
**Independent variable**				
Higher education	−0.129^***^	−0.061^**^	−0.070	0.517^*^
	(0.027)	(0.029)	(0.052)	(0.271)
Ln (Housing price)				−0.191^***^
				(0.065)
Higher education×Ln (Housing price)				−0.067^**^
				(0.030)
**Control variable**				
Demographic characteristics	Yes	Yes	Yes	Yes
Socioeconomic characteristics	Yes	Yes	Yes	Yes
Regional level variables	Yes	Yes	Yes	Yes
Year dummies	Yes	Yes	Yes	Yes
Region dummies	Yes	Yes	Yes	Yes
Intercept	Yes	Yes	Yes	Yes
Observations	12,446	16,360	12,991	41,797

(1) Robust and clustered standard errors in parentheses; (2)

* *p* < 0.1,

** *p* < 0.05,

*** *p* < 0.01.

Furthermore, based on the estimation results in Table 9, and to gain more useful information through comparison, Table 9 presents the marginal effects of higher education on people's happiness status across various regions with different housing prices. We can summarize that higher education is positively correlated with the probabilities of “extremely unhappy,” “unhappy,” and “acceptable,” while negatively correlated with the probabilities of “happy” and “extremely happy.” Meanwhile, higher education has the most positive effect on probability of “acceptable,” while has the most negative effect on probability of “extremely unhappy.” In addition, horizontal comparison between subgroup 1 and subgroup 2 in Table 11 tells us that higher education could lower people's happiness to a greater extent who live in regions with relatively high housing price. Finally, the marginal effects in subgroup 3 keep statistically insignificant for each happiness status, which once again suggests higher education has no obvious influence on people's overall happiness.

**Table 11:** Marginal effect of higher education on happiness status across various regions.

**Happiness status**	**Subgroup 1**			**Subgroup 2**			**Subgroup 3**		
	**Regions with relatively**			**Regions with relatively**			**Regions with relatively**		
	**high housing price**			**moderate housing price**			**low housing price**		
	**Marginal effect**	**Delta–method** ** standard error**	**Z** **statistics**	**Marginal effect**	**Delta–method standard error**	**Z** **statistics**	**Marginal effect**	**Delta–method** ** standard error**	**Z** **statistics**
Extremely unhappy	0.0019^***^	0.001	3.26	0.0011^**^	0.000	2.29	0.0011	0.001	1.37
Unhappy	0.0104^***^	0.002	4.68	0.0048^**^	0.002	2.19	0.0063	0.005	1.37
Acceptable	0.0359^***^	0.076	4.74	0.0166^**^	0.008	2.10	0.0192	0.014	1.34
Happy	−0.0185^***^	0.004	−4.83	−0.0079^**^	0.004	−2.05	−0.0104	0.008	−1.32
Extremely happy	−0.0297^***^	0.007	−4.55	−0.0147^**^	0.007	−2.18	−0.0161	0.012	−1.37

** *p* < 0.05 and

*** *p* < 0.01.

#### Does housing reform matters?

As mentioned earlier in this study, the historic housing reform in urban China was launched in 1998, which completely ended the welfare housing system and committed to building housing market. Obviously, the end of the welfare housing era had a great or even the greatest impact on university graduates, since they were usually assigned jobs and housing by governments and work units before that, and the housing reform dramatically boosted housing prices in Chinese cities (Chen et al., 2010; Logan et al., 2010; Wu et al., 2012). Naturally, a question arises that does housing reform launched in 1998 have impact on happiness status of highly educated people? To provide a convincing answer for above question, we would like to further conduct the empirical analysis.

Table 12 shows the difference in happiness of highly educated people in two subgroups: subgroup 1 gathers samples that graduated from universities before 1998, while subgroup 2 represents people who finished higher education after 1998. The sample scale of highly educated people in our final database is 5,804, and there are, respectively, 1,905 and 3,899 observations in above two subgroups. As expected, the mean of happiness in subgroup 1 (3.841) is significantly higher than that in subgroup 2 (3.776), which indicates market-oriented housing reform probably has negative effect on highly educated people's happiness.

**Table 12:** Difference in happiness of highly educated people in two subgroups.

**Subgroups**	**Observations**		**Difference in happiness status**						
	**Freq**.	**Percent**	**Mean**	**Std. Dev**.	**95% Conf. interval**		**Diff**.	**T Stat**.	**Sig. level**
Subgroup 1: Graduated before housing reform	1,905	32.82%	3.841	0.786	3.806	3.877	0.065	2.994	^***^
Subgroup 2: Graduated after housing reform	3,899	67.18%	3.776	0.769	3.752	3.800			

(1) Happiness status is measured on a five-point scale, ranging from 1 (extremely unhappy) to 5 (extremely happy). (2)

*** *p* < 0.01.

We then aim to empirically investigate the impact of housing reform on people's happiness status who have been highly educated. The model specification takes the following form:


(5) $$\begin{array}{l} Happiness_{it}=\alpha_{0,4}+\alpha_{1,4}{\left(Higher\;education_{after\;1998}\right)}_{it}\\ \;+\alpha_{2,4}X_{it}+\vartheta_{j}+\sigma_{t}+\mu_{it,4} \end{array}$$

where *Higher education*_*after* 1998_ represents the indicator variable of people graduating from universities after 1998, and the reference group is the people who finished higher education before 1998. In addition, other variables in Equation (5) are the same as those in previous Equation (1) in this study.

Table 13 reports the empirical result of the impact of housing reform on highly educated people's happiness. The observables are gradually added into the model from columns (1) to (4), and the coefficients of interest are significantly negative, suggesting that highly educated people who finished higher education after 1998 are more likely to become unhappier than those who graduated from universities before 1998. In other words, housing reform launched in 1998 has negatively affected highly educated people's happiness, and this is also in line with the earlier analyses in this study. Also, hypothesis 3 is thus confirmed.

**Table 13:** Impact of housing reform on happiness of highly educated people.

	**(1)**	**(2)**	**(3)**	**(4)**
	**Dependent variable: Happiness status (OPM)**			
**Independent variable (reference group: finished higher education before 1998)**				
Finished higher education after 1998	−0.113^***^	−0.105^**^	−0.087^**^	−0.086^**^
	(0.030)	(0.044)	(0.042)	(0.043)
**Control variable**				
Demographic characteristics	No	Yes	Yes	Yes
Socioeconomic characteristics	No	No	Yes	Yes
Regional level variables	No	No	No	Yes
Year dummies	Yes	Yes	Yes	Yes
Region dummies	Yes	Yes	Yes	Yes
Intercept	Yes	Yes	Yes	Yes
Observations	5,804	5,804	5,804	5,804

(1) Robust and clustered standard errors in parentheses; (2)

** *p* < 0.05 and

*** *p* < 0.01.

## Further discussion

In our previous empirical analysis process, as the key independent variable, the higher education is defined as an indicator variable and includes people have obtained bachelor degree, master degree, and Ph.D degree. In this section, we would like to separate the three types of degrees and use them as the independent variables to conduct further discussion, and the regression model takes the following form:


(6) $$\begin{array}{l} Happiness_{it}=\alpha_{0,5}+\alpha_{1,5}Bachelor_{it}+\alpha_{2,5}Master_{it}\\ \;+\alpha_{3,5}PhD_{it}+\alpha_{4,5}X_{it}+\vartheta_{j}+\sigma_{t}+\mu_{it,5} \end{array}$$

where *Bachelor*_*it*_, *Master*_*it*_, *and PhD*_*it*_ represent people who have got bachelor degree, master degree, and Ph.D degree, respectively. Other variables in Equation (6) are the same as those in previous Equation (1).

Overall, as indicated in Table 14, all the three types of degrees are negatively correlated with people's happiness status, which is consistent with the previous findings in this study. Meanwhile, different degrees of higher education have different extents of influence on happiness, and Table 15 reports the marginal effects for each of them. Specifically, all the three types of educational degrees potentially hold back people's overall happiness, and they are more likely to decrease the probability of being “extremely happy,” and increase the probability of being “acceptable.” Meanwhile, the happiness statuses of people with Ph.D degrees are generally the lowest, and people with master degrees are generally unhappier than undergraduate graduates. In summary, higher education is not conducive to people living an “extremely happy” life in urban China, instead, it potentially leads to “acceptable” lives. The higher the educational degree, the stronger the inhibitory effect of higher education on people's happiness.

**Table 14:** Relationship between different degrees and happiness status.

	**(1)**	**(2)**	**(3)**	**(4)**
	**Dependent variable:**			
	**Happiness status (OPM)**			
**Independent variable**				
Bachelor degree	−0.065^***^			−0.078^***^
	(0.021)			(0.022)
Master degree		−0.117^**^		−0.152^***^
		(0.045)		(0.044)
Ph.D degree			−0.221^**^	−0.261^**^
			(0.103)	(0.110)
**Control variable**				
Demographic characteristics	Yes	Yes	Yes	Yes
Socioeconomic characteristics	Yes	Yes	Yes	Yes
Regional level variables	Yes	Yes	Yes	Yes
Year dummies	Yes	Yes	Yes	Yes
Region dummies	Yes	Yes	Yes	Yes
Intercept	Yes	Yes	Yes	Yes
Observations	41,797	41,797	41,797	41,797

(1) Robust and clustered standard errors in parentheses; (2)

** *p* < 0.05 and

*** *p* < 0.01.

**Table 15:** Marginal effect of different degrees on happiness status.

**Educational degrees**	**Bachelor degree**			**Master degree**			**Ph.D degree**		
**Overall happiness**	**Marginal effect**	**Delta–method** ** standard error**	**Z** **statistics**	**Marginal effect**	**Delta–method standard error**	**Z** **statistics**	**Marginal effect**	**Delta–method** ** standard error**	**Z** **statistics**
Extremely unhappy	0.0018^***^	0.000	3.65	0.0035^***^	0.001	3.23	0.0060^**^	0.003	2.35
Unhappy	0.0069^***^	0.002	3.62	0.0133^***^	0.004	3.57	0.0230^**^	0.010	2.35
Acceptable	0.0188^***^	0.005	3.50	0.0367^***^	0.011	3.45	0.0630^**^	0.026	2.39
Happy	−0.0092^***^	0.003	−3.44	−0.0179^***^	0.005	−3.44	−0.0307^**^	0.013	−2.38
Extremely happy	−0.0183^***^	0.005	−3.60	−0.0358^***^	0.010	−3.48	−0.0613^**^	0.026	−2.38

(1)

** *p* < 0.05 and

*** *p* < 0.01.

Table 16 reports the relationship between different educational degrees and personal housing assets. As shown in column (1), in terms of years of being homeowner, all the three degrees are negatively correlated with it, and people with Ph.D degrees buy homes later than masters, while masters own homes later than people with bachelor degrees. Again, it suggests higher education could delay housing purchase. Columns (2) and (3), respectively, show the results of the relationship between different degrees and the amount of unpaid housing debts, unit costs of owning the housing. The regression results are in line with the earlier findings in this study, that is, higher education is significantly and positively correlated with household's unpaid housing debts, as well as the unit costs of owning the housing. In addition, according to the coefficients listed in each column, people with Ph.D degrees usually suffer greatest housing financial burdens, and master graduates are under more pressure to realize housing than people with bachelor degrees. Overall, the housing asset accumulation and housing financial burden provide new explanations for the unexpected phenomenon that higher education could not lead to happier lives in urban China.

**Table 16:** Relationship between different educational degrees and housing assets.

	**(1)**	**(2)**	**(3)**
	**Years of being homeowner**	**Logarithm of amount of unpaid housing debt**	**Logarithm of costs of owning the housing (per square meters)**
**Independent variable**			
Bachelor degree	−0.590^***^	0.500^***^	0.346^***^
	(0.198)	(0.055)	(0.041)
Master degree	−1.285^***^	1.162^***^	0.664^***^
	(0.189)	(0.224)	(0.060)
Ph.D degree	−1.971^***^	1.293^***^	0.786^***^
	(0.366)	(0.177)	(0.146)
**Control variable**			
Demographic characteristics	Yes	Yes	Yes
Socioeconomic characteristics	Yes	Yes	Yes
Regional level variables	Yes	Yes	Yes
Year dummies	Yes	Yes	Yes
Region dummies	Yes	Yes	Yes
Intercept	Yes	Yes	Yes
Observations	29,968	41,797	32,158

(1) Robust and clustered standard errors in parentheses; (2)

*** *p* < 0.01.

## Concluding remarks

Higher education is usually highly valued by society, and it is the most important way to accumulate human capital. Obviously, the notion that higher education could lead to social development is quite straightforward. However, by using the four waves of China Household Finance Survey (CHFS) data, we find that without controlling for anything, higher education has significantly positive impact on people's overall happiness, but after the covariates such as demographic characteristics, socioeconomic characteristics, and regional level variables are controlled for, higher education is negatively correlated with people's happiness status and the gap is statistically significant at the 1% level. The marginal effect analysis shows that higher education is more likely to prevent people from achieving “extremely happy” lives; instead, it probably leads to “acceptable” lives in urban China. The findings are robust to endogeneity issue, potential sample-selection bias, and functional misspecifications.

Inspired by the research findings in a bunch of existing literature that housing wealth accumulation has significantly positive impact on people's overall happiness, and based on the realities of Chinese housing market, we then try to provide some plausible explanations from the perspective of individual housing assets. The empirical results suggest that housing asset plays the mediating role in the relationship between higher education and happiness in China. Specifically, higher education has significantly negative effect on people's years of being homeowner, that is, years of schooling could delay housing purchase. As a result, higher education would evidently increase the unpaid housing debts and financial costs of housing purchase due to the soaring housing prices in Chinese cities. Meanwhile, higher education has negative effect on people's happiness in these cities with relatively high or moderate housing prices, and the higher the housing prices are, the unhappier the highly educated people would be, but in cities with relatively low housing prices, this effect is insignificant. Moreover, giving the fact that housing reform launched in 1998 in urban China had fundamentally changed housing market, as well as housing distribution system for urban employees. Thus, we have further examined the impact of housing reform on the relationship between higher education and people's happiness status, and the result shows that market-oriented housing reform that launched in 1998 has negative impact on highly educated people's happiness. Lastly, we further divide the highly educated people into three groups, including people with bachelor degree, master degree, and Ph.D degree, and we find that Ph.D graduates are the relatively unhappiest people compared to bachelors or masters.

In summary, higher education could not lead to happier lives in urban China, although it could usually lead to better job opportunities, higher income, and social classes (Frey and Stutzer, 2002; Haveman and Smeeding, 2006; Guardiola and Guillen-Royo, 2015). We would like the define this finding as “education-happiness paradox,” and the housing asset accumulation and housing financial burden provide new explanations for this unexpected phenomenon. Obviously, this is not a good signal for the society. Because compared to housing wealth accumulation, if human capital investment could not improve quality of lives, but reduce people's happiness, on the one hand, it may breed an impetuous social atmosphere and weaken people's motivation to get higher education to some extent; on the other hand, it would further overheat the real estate markets, which would cause more serious social issues and far-reaching social problems. Therefore, Chinese government should continue to take effective measures to suppress housing prices rising too fast and make the real estate market return to rationality and provide some more necessary policy supports for highly educated people to achieve housing dreams. These are helpful to guide people to correct outlooks on housing wealth and improve people's happiness.

## Data availability statement

The original contributions presented in the study are included in the article/supplementary material, further inquiries can be directed to the corresponding author.

## Ethics statement

Ethical review and approval was not required for the study on human participants in accordance with the local legislation and institutional requirements. Written informed consent from the patients/ participants or patients/participants legal guardian/next of kin was not required to participate in this study in accordance with the national legislation and the institutional requirements.

## Author contributions

All authors listed have made a substantial, direct, and intellectual contribution to the work and approved it for publication.

## Funding

Funds are received from National Natural Science Foundation of China (72104109, 71974003, and 72203167) and University Philosophy and Social Science Research Project of Jiangsu Province (2022SJYB0122).

## Conflict of interest

The authors declare that the research was conducted in the absence of any commercial or financial relationships that could be construed as a potential conflict of interest.

## Publisher's note

All claims expressed in this article are solely those of the authors and do not necessarily represent those of their affiliated organizations, or those of the publisher, the editors and the reviewers. Any product that may be evaluated in this article, or claim that may be made by its manufacturer, is not guaranteed or endorsed by the publisher.

## Footnotes

^1^Available at: https://ourworldindata.org/human-development-index.

^2^Available at: https://worldhappiness.report.

^3^Available at: http://hdr.undp.org/en/2019-report.

^4^Data source: The World Bank (Available at: https://data.worldbank.org/indicator/SE.TER.ENRR).

^5^Available at: https://chfser.swufe.edu.cn/datasso/Home/Login.

^6^Available at: http://www.stats.gov.cn/.
